# Polyomavirus T Antigen Induces *APOBEC3B* Expression Using an LXCXE-Dependent and TP53-Independent Mechanism

**DOI:** 10.1128/mBio.02690-18

**Published:** 2019-02-05

**Authors:** Gabriel J. Starrett, Artur A. Serebrenik, Pieter A. Roelofs, Jennifer L. McCann, Brandy Verhalen, Matthew C. Jarvis, Teneale A. Stewart, Emily K. Law, Annabel Krupp, Mengxi Jiang, John W. M. Martens, Ellen Cahir-McFarland, Paul N. Span, Reuben S. Harris

**Affiliations:** a Department of Biochemistry, Molecular Biology and Biophysics, Masonic Cancer Center, Institute for Molecular Virology, University of Minnesota, Minneapolis, Minnesota, USA; b Department of Radiation Oncology, Radiotherapy and OncoImmunology Laboratory, Radboud University Medical Center, Nijmegen, The Netherlands; c Department of Microbiology, University of Alabama at Birmingham, Birmingham, Alabama, USA; d Department of Neuroimmunology, Biogen, Cambridge, Massachusetts, USA; e Department of Medical Oncology, Cancer Genomics Netherlands, Erasmus MC Cancer Institute, Erasmus University Medical Center, Rotterdam, The Netherlands; f Howard Hughes Medical Institute, University of Minnesota, Minneapolis, Minnesota, USA; University of California, Irvine; Harvard Medical School; University of Cambridge; Universidade Federal do Rio de Janeiro

**Keywords:** APOBEC3B, RB/E2F pathway, polyomavirus, virus evolution

## Abstract

APOBEC3B is a single-stranded DNA cytosine deaminase with beneficial innate antiviral functions. However, misregulated APOBEC3B can also be detrimental by inflicting APOBEC signature C-to-T and C-to-G mutations in genomic DNA of multiple cancer types. Polyomavirus and papillomavirus oncoproteins induce APOBEC3B overexpression, perhaps to their own benefit, but little is known about the cellular mechanisms hijacked by these viruses to do so. Here we investigate the molecular mechanism of APOBEC3B upregulation by the polyomavirus large T antigen. First, we demonstrate that the upregulated APOBEC3B enzyme is strongly nuclear and partially localized to virus replication centers. Second, truncated T antigen (truncT) is sufficient for APOBEC3B upregulation, and the RB-interacting motif (LXCXE), but not the p53-binding domain, is required. Third, genetic knockdown of RB1 alone or in combination with RBL1 and/or RBL2 is insufficient to suppress truncT-mediated induction of *APOBEC3B*. Fourth, CDK4/6 inhibition by palbociclib is also insufficient to suppress truncT-mediated induction of *APOBEC3B*. Last, global gene expression analyses in a wide range of human cancers show significant associations between expression of *APOBEC3B* and other genes known to be regulated by the RB/E2F axis. These experiments combine to implicate the RB/E2F axis in promoting *APOBEC3B* transcription, yet they also suggest that the polyomavirus RB-binding motif has at least one additional function in addition to RB inactivation for triggering *APOBEC3B* upregulation in virus-infected cells.

## Abstract (precis)

The APOBEC3B DNA cytosine deaminase is overexpressed in many different cancers and correlates with elevated frequencies of C-to-T and C-to-G mutations in 5′-TC motifs, oncogene activation, acquired drug resistance, and poor clinical outcomes. The mechanisms responsible for APOBEC3B overexpression are not fully understood. Here, we show that the polyomavirus truncated T antigen (truncT) triggers APOBEC3B overexpression through its RB-interacting motif, LXCXE, which in turn likely modulates the binding of E2F family transcription factors to promote *APOBEC3B* expression. This work strengthens the mechanistic linkage between active cell cycling, APOBEC3B overexpression, and cancer mutagenesis. Although this mutational mechanism damages cellular genomes, viruses may leverage it to promote evolution, immune escape, and pathogenesis. The cellular portion of the mechanism may also be relevant to nonviral cancers, where genetic mechanisms often activate the RB/E2F axis and APOBEC3B mutagenesis contributes to tumor evolution.

## INTRODUCTION

Genetic diversity is key to virus replication, pathogenesis, and transmission, and particularly for escape from adaptive immune responses in vertebrate species (1–3). Each virus has evolved to maintain an optimized level of genetic diversity for its own unique life cycle through various mechanisms, with some viruses having high mutation rates and others much lower mutation rates, notably the dsDNA viruses (4, 5). Recently, it has been concluded that the genome compositions of multiple DNA tumor viruses, including high-risk human papillomavirus (HPV) types and BK polyomavirus (BKPyV), have been shaped by long-term interactions with the innate, antiviral APOBEC deaminases (6–9). It has also been suggested that acutely occurring mutations by these enzymes in the major capsid gene of polyomaviruses promote antibody escape during polyomavirus-associated nephropathy and progressive multifocal leukoencephalopathy (10). The overlap between these disease variants and oncogenic enzymes is striking especially in light of growing evidence linking BKPyV infection and a subset of urothelial carcinomas with high levels of APOBEC-signature mutations (11).

Several APOBEC enzymes, including APOBEC3B (A3B), bind 5′-TC dinucleotide motifs in single-stranded DNA and catalyze the hydrolytic conversion of cytosine to uracil (12, 13). Left unrepaired, uracil lesions can serve as the templates for new DNA synthesis and directly result in C-to-T mutations. Alternatively, if the uracil base is excised by cellular uracil DNA glycosylase 2 (UNG2), then the resulting abasic site becomes noninstructional and may trigger cellular DNA polymerases to insert an adenine opposite the lesion, except for REV1, which tends to incorporate either adenine or cytosine. Thus, APOBEC-catalyzed DNA deamination of 5′-TC motifs results in both C-to-T and C-to-G mutations (a signature frequently expanded to include the 3'-nucleobases A and T and referred to in the context of trinucleotide motifs 5′-TCA and 5′-TCT). An additional hallmark of virus mutagenesis by APOBEC enzymes is a bias toward occurring on the template of lagging-strand DNA replication (14–16). A likely mechanistic relationship with single-stranded DNA replication intermediates is supported by similar correlations in model yeast and Escherichia coli experiments (17, 18).

Human cells have the potential to express up to nine active DNA cytosine deaminases (AID, APOBEC1, and A3A/B/C/D/F/G/H) (19–22). Seven of these enzymes prefer 5′-TC motifs in single-stranded DNA, whereas AID uniquely prefers 5′-RC and APOBEC3G (A3G) prefers 5′-CC. A3B is the most likely APOBEC family member to contribute to the mutagenesis and evolution of small DNA tumor viruses because it is specifically upregulated by viral oncoproteins. For high-risk HPV types, the oncoproteins E6 and E7 have been implicated through various pathways (23–26). For polyomaviruses, including JC, BK, and Merkel cell (JCPyV, BKPyV, and MCPyV, respectively), the large T antigen (TAg) is sufficient for A3B upregulation through a yet-to-be determined mechanism (6). However, the considerable functional overlap of these proteins, RB inactivation by E7 and TAg and p53 inactivation by E6 and TAg, may indicate limited pathways for A3B modulation by viruses (27, 28). Here we investigate the molecular mechanism by which polyomaviruses promote the transcriptional upregulation of *A3B* with results converging on the cellular RB/E2F pathway, which is often deregulated in cancer.

## RESULTS

### Visualization of endogenous APOBEC3B protein in polyomavirus-infected cells.

**FIG 1:**
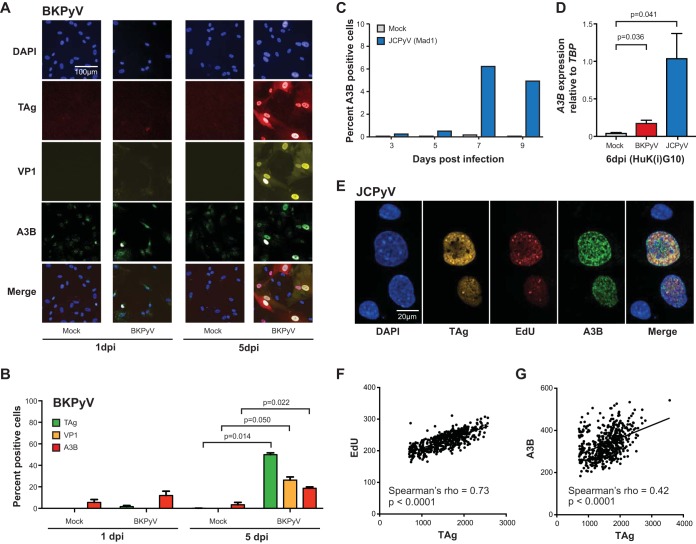
Visualization and quantification of A3B expression in PyV-infected cells. (A and B) Immunofluorescent images and quantification of TAg, VP1, and A3B in BKPyV-infected HuK(i)G10 cells at 1 and 5 dpi (significance determined using Welch’s two-tailed *t* test; *n* = 2 biological replicates). (C) Time course of *A3B* mRNA levels in JCPyV (Mad1 strain) versus mock-infected HuK(i)G10 cells. (D) RT-qPCR quantification of *A3B* transcripts in mock-, BKPyV-, and JCPyV (Mad1)-infected HuK(i)G10 cells at 6 dpi (significance determined by Welch’s two-tailed *t* test; *n* = 3 technical replicates). (E) High-resolution immunofluorescent microscopy images of DAPI, A3B, EdU, and TAg in HuK(i)G10 cells infected with JCPyV (Mad1 strain). (F and G) Correlation coefficients and *P* values for EdU and A3B levels versus T antigen intensity in >100 cell images from a single experiment similar to that in panel E.

A3B induction by polyomaviruses has been shown at the mRNA level by RT-qPCR and at the protein level by immunoblotting in primary renal proximal epithelial cells (RPTECs) (6). To extend these results to other relevant cell types, RT-qPCR and immunofluorescent microscopy were used to ask whether polyomavirus infection causes a general pan-nuclear upregulation of A3B enzyme and/or localization to discrete subnuclear regions such as virus replication centers. Immortalized human kidney [HuK(i)G10] cells were infected with BKPyV (Dunlop strain) and JCPyV (MAD1 strain) and subjected to analyses at various days postinfection (dpi). Infected cells have enlarged nuclei and robust expression of TAg and VP1 at 3 to 5 dpi (Fig. 1A). A3B expression was more variable but still clearly and significantly increased after infection with either virus compared to mock-infected controls (Fig. 1A to D). Generally, JCPyV is regarded to have slower replication dynamics than BKPyV (Dunlop), so initial JCPyV infections were run out in a time course showing peak A3B expression at 7 dpi (Fig. 1C). Across these experiments, JCPyV-infected HuK(i)G10 cells showed a greater differential expression of A3B mRNA and protein compared to mock-treated cells (Fig. 1B to D).

JCPyV-infected cells were also analyzed 7 dpi by high-resolution immunofluorescent microscopy for expression of A3B and viral proteins and for formation of virus replication foci. Cells were stained for DAPI, TAg, A3B, and EdU with virus replication centers appearing as brightly stained puncta positive for both TAg and EdU (representative images in Fig. 1A and E) (29). In infected cells, A3B is strongly induced with a pan-nuclear staining pattern that is sometimes coincident with EdU-positive virus replication foci. Incorporation of EdU into active replication foci is highlighted by strong positive correlations with TAg stain intensity, as expected, whereas A3B showed weaker but still significantly positive correlations (Fig. 1F and G). These data indicate that A3B upregulation may be a general property of polyomavirus infection and that A3B may access at least a subset of virus replication centers.

### APOBEC3B upregulation by polyomavirus large T antigen requires the canonical RB-interacting motif LXCXE.

**FIG 2:**
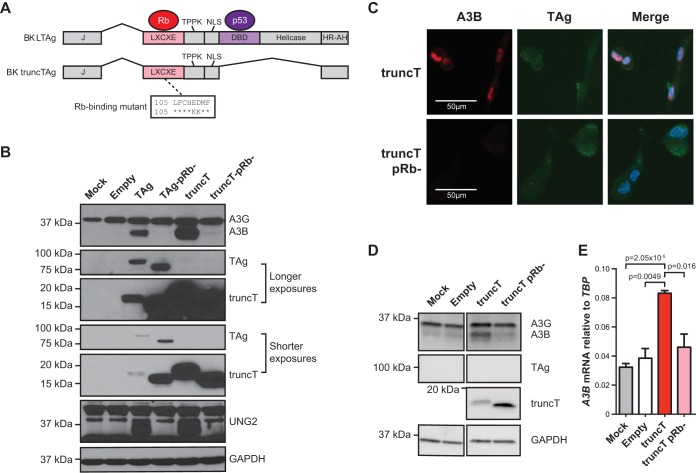
RB-binding domain is necessary for A3B induction by polyomavirus T antigen. (A) Diagram of BKPyV T antigen isoforms and the LXCXE mutant used here. (B) Immunoblots for the indicated proteins in RPTECs transduced with a lentiviral vector expressing LTAg, truncT, the indicated mutants, or nothing (empty). Mock-transduced cells were analyzed in parallel as an additional control. (C) Immunofluorescent microscopy images for truncT, truncT LXCXE mutant, and A3B in transduced RPTECs. (D and E) Immunoblots and RT-qPCR results for MCF10A cells transduced with the indicated constructs as in panel B (mean and SEM shown in panel E; *n* = 3 biological replicates; *P* value determined by Welch’s two-tailed *t* test).

Based on the results above and our previous studies (6), multiple polyomaviruses have the conserved capacity to upregulate A3B expression in primary and immortalized kidney epithelial cells through the functions of large (L) TAg. To investigate the LTAg domains responsible for A3B induction, and thus also implicate associated cellular factors, we tested a naturally occurring splice variant of BKPyV LTAg, known as truncT, which lacks the DNA-binding and helicase domains essential for p53 neutralization (30–32) (schematic in Fig. 2A). In parallel, we also assessed derivatives of LTAg and truncT with a disrupted LXCXE motif, which is required for inhibiting the tumor suppressor protein RB1 as originally shown for SV40 TAg (33). RPTECs were transduced with lentiviruses expressing an empty multiple cloning site as a negative control, BKPyV LTAg as a positive control, BKPyV truncT, and RB-binding site mutant derivatives; incubated 3 days; and assessed by immunoblotting and fluorescence microscopy. Mock-transduced cells express low levels of A3G, and transduction with empty lentivirus causes a modest increase in this protein and also raises A3B levels to faintly detectable levels (Fig. 2B). In contrast, both LTAg and truncT induce expression of A3B and UNG2, a known target of the RB-E2F axis (34), and all induction for both of these proteins is eliminated by two amino acid substitutions shown to abrogate RB binding in SV40 TAg (LFCHED to LFCHKK) (33) (Fig. 2B). The LTAg and truncT mutants invariably migrate faster than the corresponding wild-type proteins during SDS-PAGE, which is likely due to a charge differential caused by the two amino acid substitutions. Immunofluorescent microscopy images also show truncT-mediated induction of nuclear A3B but not by the RB-binding mutant derivative (Fig. 2C).

To date, many aspects of A3B regulation and function have been determined using normal-like and cancerous mammary epithelial cell lines due to higher capacities for genetic manipulation over primary cells and greater relevance to cancer (35, 36). To ask whether TAg induction of A3B also occurs in one of these more tractable systems, the normal-like mammary epithelial cell line MCF10A was transduced with constructs expressing BKPyV truncT or the RB-binding mutant and analyzed as described above. Both immunoblotting and RT-qPCR yielded similar results with truncT but not the RB-binding mutant causing A3B induction (Fig. 2D and E). Thus, polyomavirus T antigen appears to possess a conserved, LXCXE-dependent capacity to induce A3B in different epithelial cell types.

### TP53 inactivation is dispensable for *APOBEC3B* induction.

**FIG 3:**
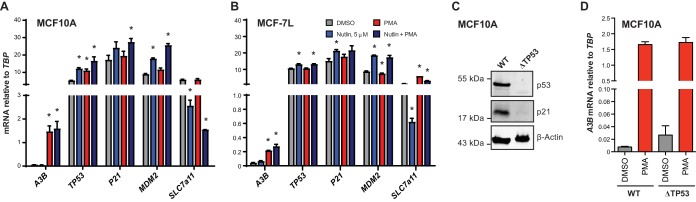
Inactivation of p53 does not affect A3B expression. (A and B) Bar plots of RT-qPCR measurements of relevant genes in MCF10A (A) and MCF7L (B) cells treated with DMSO, 5 µM nutlin, PMA, or nutlin + PMA. Statistically significant changes by Student’s *t* test (*P* < 0.05) are noted by an asterisk (mean and SEM; *n* = 3 technical replicates). (C) Immunoblots of WT and *TP53* KO MCF10A cell lines. (D) RT-qPCR results showing the effects of PMA treatment on *A3B* mRNA levels in WT and *TP53* KO MCF10A cell lines.

The aforementioned data comparing LTAg and truncT simultaneously implicate RB1 and demonstrate that p53 inhibition is not required for A3B induction because truncT completely lacks the p53 binding domain (Fig. 2). To further ask whether p53 inactivation might influence *A3B* gene expression, we quantified *A3B* mRNA levels in two cell lines that have been used to study A3B regulation, MCF10A and the human estrogen receptor-positive breast cancer cell line MCF-7L (above and references 35 to 38). Each cell line was treated with either DMSO or 5 µM nutlin, which is a drug that protects p53 from MDM2-mediated degradation (39). As controls, mRNA levels were quantified for two genes activated by p53 (*P21*, *MDM2*) (40–43) and one gene repressed by p53 (*SLC7A11*) (44, 45). Respectively, the expression of these genes was derepressed or repressed by nutlin treatment (Fig. 3A and B). In comparison, neither steady-state nor PMA-induced *A3B* mRNA levels were changed by nutlin (Fig. 3A and B). Moreover, Cas9-mediated knockout of *TP53* in MCF10A cells also caused no significant effect on basal or PMA-induced *A3B* expression levels (Fig. 3C and D). These data combine to indicate that p53 by itself has no significant role in the either the PMA-induced pathway or basal-state transcriptional regulation of *A3B*, discouraging our original hypothesis (23) and conflicting with published data (38) (see Discussion).

### RB-family knockdown is insufficient to induce *APOBEC3B* expression.

**FIG 4:**
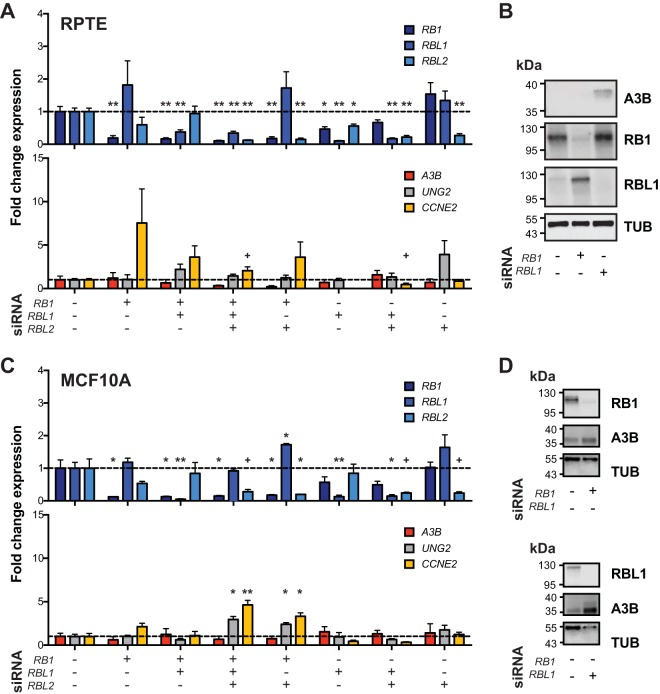
Modulation of RB family genes and A3B regulation. (A and C) Bar plots of RT-qPCR quantification of *RB*-family mRNAs (top) and predicted responsive genes, *A3B*, *UNG2*, and *CCNE2* (bottom), in RPTEC and MCF10A cells with siRNA-mediated KD of *RB*-family genes. *P* values for each siRNA combination compared to control were calculated using Welch’s two-tailed *t* test and were indicated using the following symbols: +, *P* < 0.1; *, *P* < 0.05; **, *P* < 0.01; *n* = 3 biological replicates. (B and D) Immunoblots for A3B, RB1, and RBL1 in RPTEC and MCF10A cells following treatment with the indicated siRNA.

RB1 is arguably the most widely studied target of the LXCXE motif of viral proteins such as HPV E7, adenovirus E1A, and polyomavirus LTAg (27, 31, 32, 46). However, the related pocket proteins RBL1 (p107) and RBL2 (p130) also have an LXCXE-binding motif, are similarly targeted and inactivated by LTAg and truncT, and may be involved in A3B regulation (47–50). The aforementioned viral proteins bind to the hypophosphorylated forms of RB1, RBL1, and RBL2, which inhibits phosphorylation by cyclin-dependent kinases (CDKs) and leads to an accelerated cell cycle in part by deregulation of E2F transcriptional activities. To investigate the roles of RB1, RBL1, and RBL2 in A3B transcriptional regulation, a series of knockdown experiments was done with siRNAs targeting each of these factors in RPTECs and MCF10A cells (Fig. 4A to D). RT-qPCR showed that >75% knockdown was achieved for each targeted gene (upper panels in Fig. 4A and C). As controls, *CCNE2* was upregulated upon *RB1* knockdown and *UNG2* was moderately upregulated by *RBL2* knockdown (lower panels in Fig. 4A and C). However, no combination of siRNAs resulted in significant upregulation of *A3B* mRNA levels (lower panels in Fig. 4A and C). Knockdown of RB1 and RBL1 was validated at the protein level, but RBL2 could not be clearly discerned with available commercial antibodies (Fig. 4B and D). In contrast to the RT-qPCR results, protein-level A3B expression did appear to be elevated upon RBL1 knockdown. These results suggest that depletion of each RB family member alone or in combination is insufficient to significantly upregulate *A3B* mRNA levels, at least in these two different normal-like cell types where *A3B* is induced by TAg and truncT. However, the observed upregulation at the protein level in RBL1-depleted cells raises the possibility of an additional layer of regulation that may be posttranscriptional. These results also suggest that truncT may have at least one additional activity mediated by its LXCXE motif that contributes to *A3B* upregulation.

### Pharmacological inhibition of CKD4/6 does not alter *APOBEC3B* expression.

**FIG 5:**
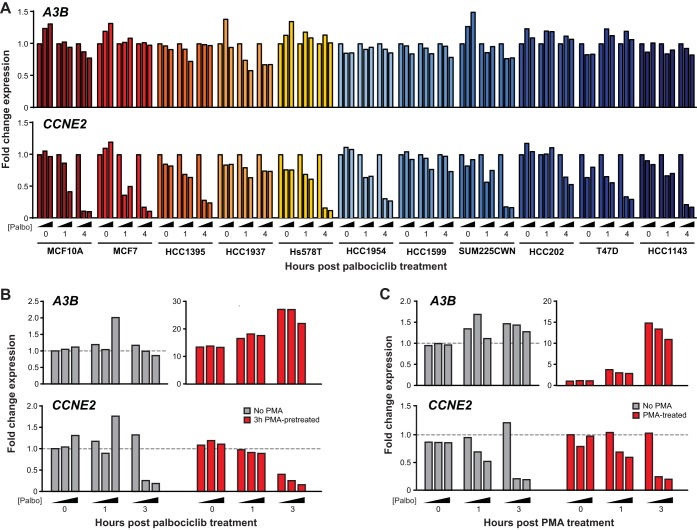
Palbociclib treatment of cancer cell lines and A3B expression. (A) RT-qPCR quantification of *A3B* and *CCNE2* mRNA expression in the indicated cell lines treated with 0, 0.5, or 2.5 µM palbociclib for 0, 1, or 4 h. (B) RT-qPCR quantification of *A3B* and *CCNE2* expression in MCF10A cells pretreated with 0 ng/ml or 25 ng/ml PMA for 3 h prior to treatment with 0, 0.5, or 2.5 µM palbociclib for 0, 1, and 3 h. (C) RT-qPCR quantification of *A3B* and *CCNE2* expression in MCF10A cells pretreated with 0, 0.5, or 2.5 µM palbociclib for 30 min and then treated with 0 ng/ml or 25 ng/ml PMA for 0, 1, and 3 h.

Palbociclib is a selective inhibitor of CDK4 and CDK6, which are kinases that function normally to phosphorylate pRB, prevent binding to E2F transcription factors, and stimulate the expression of many genes involved in cell cycle progression (51–53). To corroborate the knockdown experiments above, we treated a panel of transformed cell lines with palbociclib and quantified mRNA expression levels over time. This panel of cell lines was constructed based on *A3B* expression, ranging from low to high (54, 55), *TP53* status, and ability to phosphorylate RB. As a positive control for palbociclib efficacy, we analyzed expression of *CCNE2*, which encodes cyclin E2, promotes entry into S phase, and is a known CDK4/6-RB-regulated gene (56, 57). The majority of cell lines showed a dose- and time-responsive decrease in *CCNE2* mRNA expression (Fig. 5A). This effect was minimal in HCC1937 and HCC1599 cells, which are known to display decreased RB phosphorylation (58, 59). In contrast, none of the palbociclib-treated cell lines showed a reproducible or significant change in *A3B* mRNA expression. In addition, MCF10A cells were treated with PMA to induce *A3B* mRNA expression by the PKC/ncNF-κB pathway, and again, palbociclib had little effect (palbociclib added post- or pre-PMA addition in Fig. 5B and C). These results combine to indicate that the kinase activity of CDK4 and CDK6 is dispensable for *A3B* expression in multiple different cell lines.

### Tumor transcriptome analyses support involvement of the RB pathway in *APOBEC3B* regulation.

**FIG 6:**
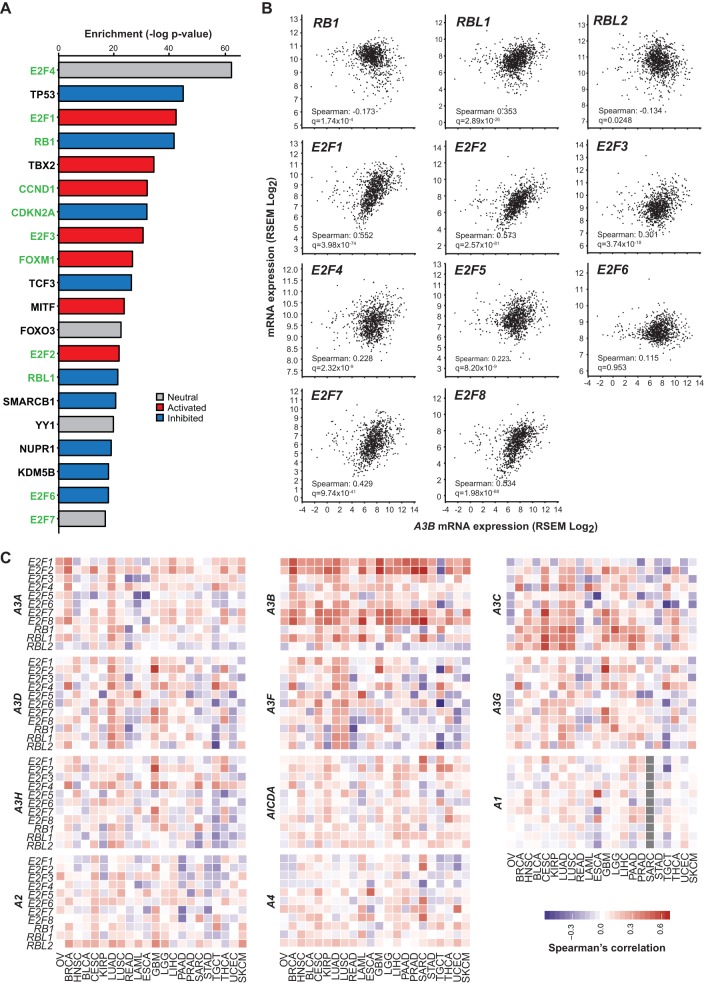
Evidence for *A3B* regulation by the RB/E2F pathway in tumors. (A) Top 20 hits from IPA enrichment analysis of upstream transcriptional regulators of *A3B* in TCGA breast cancer with RB-pathway/cell cycle-related genes highlighted in green. Negative logs of enrichment *P* values are shown in the bar graph on the right colored by predicted activation (red) or inhibition (blue) of the specific transcription factor. (B) Scatter plots showing the correlation between *A3B* mRNA levels and transcription factors in the RB pathway with Spearman’s correlation coefficient and *q* value reported in the lower left corner of each subpanel. (C) Spearman’s correlation coefficient values for all APOBEC-family members against RB pathway transcription factors across 22 cancers ordered by hierarchal clustering. Negative correlations are shown in blue, positive correlations are shown in red, and no data is represented by grey.

We next used bioinformatics approaches to mine TCGA data and assess global correlates with *A3B* mRNA expression in human tumors. First, we conducted pathway analysis using all genes with significant positive correlations between *A3B* expression in the TCGA breast tumor cohort. This analysis revealed that 11 of the top 20 significantly enriched upstream transcription factors contributing to this expression pattern are part of the CDK4/6-cyclin D-RB-E2F axis (green-labeled genes in Fig. 6A). These regulatory factors were either significantly activated or inhibited, generally corresponding with known functions, with the net outcome being accelerated cell cycling (respectively, red and blue bars in Fig. 6A). Upon closer pairwise examination of effectors in this signal transduction pathway, *A3B* mRNA expression has the strongest positive correlations with expression of *RBL1*, *E2F1*, *E2F2*, *E2F7*, and *E2F8* (Fig. 6B).

Last, we expanded this expression correlation analysis to include 22 different tumor types in TCGA and all 11 *APOBEC* family members. This global approach further highlighted strong correlations between *A3B* and expression of *E2F1*, *E2F2*, *E2F7*, and *E2F8* and indicated that the association between *A3B*, this signal transduction pathway, and the cell cycle is evident in many cancer types (Fig. 6C). Heat map intensities also indicated that *A3B* is the only *APOBEC* family member that positively and globally correlates with activation of the RB-E2F axis. To further reconcile our experimental and bioinformatics data sets, we used the meta-analysis regulatory data from TargetGeneReg (http://www.targetgenereg.org/) to compare the regulation of *A3B* with known cell cycle-related genes (60). These results, summarized in Table 1, further indicate that the *A3B* mRNA expression profile is consistent with that of a cell cycle-regulated gene that becomes upregulated during the G_2_/M phase, which is similar to *FOXM1* and distinct from *UNG2*, *CCNE2*, and *A3C* (the last being an *APOBEC3* family member likely to be regulated by p53).

**TABLE 1:** Cell cycle analysis of *A3B* and other cell cycle-regulated genes

Type of value	Value for gene:				
	*APOBEC3B*	*APOBEC3C*	*CCNE2*	*FOXM1*	*UNG2*
Chromosome	22	22	8	12	12
p53 expression score	−4	14	−14	−17	−14
No. of cell cycle data sets	2	1	5	2	5
G_1_/S or G_2_/M	G_2_/M	0	G_1_/S	G_2_/M	G_1_/S
p53 target	No	Yes	No	No	No
Cell cycle gene	Yes	No	Yes	Yes	Yes
DREAM target	Yes	No	No	Yes	Yes
MMB-FoxM1 target	No	No	No	No	No
RB-E2F target	No	No	Yes	No	Yes

## DISCUSSION

In this study, we investigate the mechanism of *A3B* upregulation by polyomavirus T antigen through analyses of separation-of-function mutants, genetic knockdowns, pharmacologic treatments, and transcriptomic data. We use high-resolution fluorescence microscopy to show that polyomavirus infection causes *A3B* upregulation and protein accumulation in the nuclear compartment with the potential to access viral replication foci. Second, we show that the LXCXE motif of LTAg and truncT, which is well known to inhibit the tumor suppressor RB, is essential for *A3B* upregulation, whereas the p53-binding domain is dispensable. Further investigation into this pathway using genetic and pharmacologic treatments indicate that solely perturbing RB family members (RB, RBL1, and RBL2) or kinases responsible for their phosphorylation (CDK4 and CDK6) is insufficient to cause *A3B* mRNA upregulation. However, bioinformatics analyses of tumor expression data show strong global correlations between *A3B* mRNA expression and expression of other genes regulated by the RB-E2F signaling pathway, including several members of the E2F family of transcription factors.

**FIG 7:**
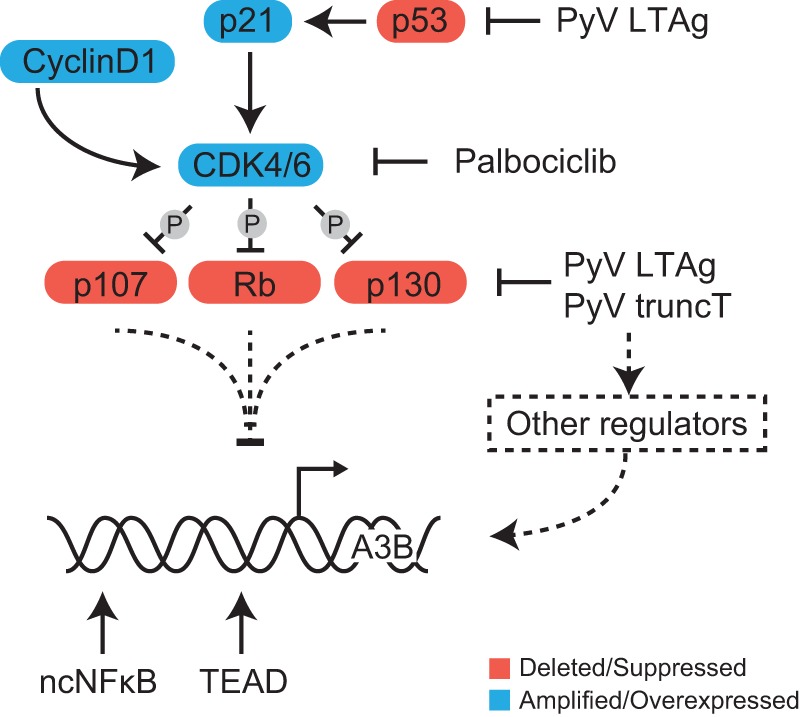
Model for *A3B* transcriptional regulation. Schematic of the cell cycle-related proteins affected by T antigen and drug treatments used in this study with implications for *A3B* transcriptional regulation. Solid lines represent established processes, and dashed lines represent regulatory interactions/pathways implicated by our studies.

Additional analysis of cell cycle-regulatory networks using the TargetGeneReg database suggest that *A3B* might be a late cell cycle gene repressed by the RB/E2F family members associated with the DREAM complex in quiescence and activated by other transcription factors. Suppression by the DREAM complex is supported by the mild upregulation of A3B protein observed upon RBL1 knockdown in this study. One putative activating transcription factor is FOXM1, which was significantly enriched as an upstream regulator of *A3B* and is known to activate genes in late G_2_/M (61). Interestingly, in lymphoblastoid B cells, FOXM1 has been reported to frequently cooccupy NF-κB binding sites and form protein complexes with NF-κB transcription factors, which have been implicated in *A3B* regulation (36, 62). Taken together, these results indicate that the RB/E2F pathway, which is commonly modified in cancer, likely contributes to *A3B* overexpression observed in virus infections and in different tumor types, but additional unknown signals are also likely to be required for full induction (model in Fig. 7).

Our original studies with HPV and A3B led us to propose a model in which p53 represses *A3B* transcription and that p53 inactivation by the viral oncoprotein E6 or by genetic mutation results in derepression of *A3B* transcription (23). This transcription repression model is consistent with strong correlations in tumors and cell lines between *A3B* overexpression and *TP53* inactivation (54). A recent study confirmed these correlations and used pharmacologic and genetic approaches to provide further support for such a model (38). However, three different results in the cell-based systems presented here do not support a dominant role for p53 in *A3B* repression. Specifically, the p53 binding domain of BKPyV is dispensable for *A3B* upregulation, nutlin treatment has no effect on basal or induced *A3B* expression, and p53 knockout alone fails to induce *A3B* expression (Fig. 2 and 3). Moreover, another recent study showed that p53 inactivation renders cells more permissive for A3B mutagenesis (55). Therefore, we disfavor a transcriptional role and now favor a “tolerance model” in which p53 inactivation (genetic, epigenetic, or viral) is required for cells to be able to tolerate the increased levels of DNA damage caused by A3B overexpression. This model also explains why somatic *TP53* mutations were identified as a significant global correlate with *A3B* overexpression in cancer (11).

The RB-E2F signaling axis is one of the most frequently mutated pathways in cancer, which contributes to several hallmarks of cancer by deregulating the cell cycle (63). RB inactivation is also a common target for viral genes in order to promote the survival of infected cells. The integration and continued expression of viral oncogenes in the host genome are common characteristics of virus-associated tumors. For example, HPV-positive tumors frequently express the viral E7 oncoprotein, which also has an LXCXE motif, and this is thought to be critical for tumor development (64, 65). These tumors also tend to have a high burden of APOBEC-associated mutations on the DNA strand that serves as the template for lagging-strand replication, which is synthesized during the S phase of the cell cycle (11, 14, 17). Although some of these effects have been explained by perturbations to the p53 pathway, other pathways affected by E6 have also been shown to alter *A3B* gene expression, such as those regulated by the TEAD and ZNF384 transcription factors (23, 25, 36, 38). It is therefore not surprising in hindsight that expression of the antiviral enzyme A3B is also induced upon disruption of the RB-E2F pathway. This idea is supported by a study showing that high-risk HPV E7 is capable of upregulating A3B (26). An additional study also found that elevated *A3B* expression is significantly correlated with proliferative features in breast cancer (66). Last, the LXCXE motif may be acting through another cellular signaling pathway. For instance, the LXCXE of various viral proteins triggers activation of the antiviral cGAS-STING pathway, which could also contribute to *A3B* transcriptional regulation (67).

All of these results combine to indicate that *A3B* transcriptional regulation is complex and governed by multiple pathways and different transcription factors. Perhaps linkage to the cell cycle evolved to prevent potentially oncogenic mutations of the host genome during normal cellular DNA replication or, alternatively, to maximize antiviral responses during particularly susceptible cell cycle stages. Further experiments using mutant viral oncogenes as molecular probes, such as T antigen, E6, and E7, are likely to continue to provide valuable insights into the regulation of *A3B* and lead to a greater understanding of its roles in tumorigenesis, virus evolution, and antiviral immunity.

## MATERIALS AND METHODS

### Cell lines, culture conditions, and lentivirus production.

Primary renal proximal tubule epithelial cells (RPTECs; Lonza) were grown in REGM (Lonza). MCF10A cells were grown in MEGM (Lonza) containing penicillin (100 U/ml) and streptomycin (100 μg/ml). HuK(i)G10 cells were grown in RenaLife epithelial medium (Lifeline Cell Technologies) with 5% FBS. MCF7 and derivative cell lines were grown in Richter’s modification medium containing 5% fetal bovine serum, penicillin (100 U/ml), streptomycin (100 μg/ml), and 11.25 nM recombinant human insulin. All cell lines were grown at 37°C in a 5% CO_2_ incubator. Lentiviruses expressing TAg and mutant derivatives were produced in 293T cells and transduced into RPTECs as described previously (23).

### Antibodies.

Large T and truncT forms of BKPyV T antigen were detected using pAb416 (30). JCPyV large T antigen was detected using PAB2000 (68). A Harris lab custom rabbit anti-human A3B monoclonal antibody, 5210.87.13, was used in immunoblotting assays and in high-resolution immunofluorescent microscopy experiments with JCPyV-infected HuK(i)G10 cells (69). Santa Cruz sc-130688 was used for A3B quantification and lower-resolution microscopy of BKPyV- and JCPyV-infected HuK(i)G10 cells (69). UNG2 was detected using the Abcam antibody ab23926. RB1 was detected using Santa Cruz sc-102, and RBL1 was detected using Cell Signaling 89798, whereas RBL2 could not be detected in immunoblots with Abcam antibody ab71143. TP53 (p53) was detected using clone DU-1 (Santa Cruz SC-126), p21 using CST clone 1201 (CST no. 2947), and beta-actin using CST clone 13E5 (CST no. 4970).

### RNA isolation, RT-qPCR, and immunoblots.

Total RNA was harvested by removal of medium and resuspension in TRIzol (Thermo Fisher), and purification was done per the manufacturer’s protocol. RT-qPCR was used to quantify *A3B* and *UNG2* transcripts in siRNA experiments as described previously (11, 23) and these methods were adapted for *CCNE2*. Protein lysates from virus and siRNA experiments were harvested at 3 or 7 dpi or postransduction, quantified, and immunoblotted as described previously (70). Data were plotted and *t* tests were calculated using GraphPad Prism 7.

### Immunofluorescent microscopy experiments.

HuK(i)G10 kidney cells were seeded at 6,000 cells/well in a 96-well plate. Twenty-four hours later, infection with JCPyV was performed as described, and then cells were collected 7 dpi. Infected cells were incubated with EdU (Click-iT Plus EdU Alexa Fluor 647 imaging kit; Thermo Fisher Scientific) for 15 min and incubated with CSK buffer (10 mM HEPES-KOH, pH 7.4; 300 mM sucrose; 100 mM NaCl; 3 mM KCl; 0.5% Triton X-100) (71) for 2 min on ice. Cells were then fixed in 4% PFA for 10 min followed by permeabilization with 0.5% Triton X-100 for 20 min. For EdU detection, the Click-iT reagent was added for 30 min in the dark according to the manufacturer’s protocol and washed three times with PBS. Samples were incubated with BlockAid blocking solution (Thermo Fisher) for 1 h at room temperature. T antigen, VP1, and A3B staining was performed using the aforementioned antibodies at 1:1,000, 1:1,000, and 1:100 (1:50 for sc-130688) dilutions in BlockAid, respectively, overnight at 4°C followed by staining with the secondary antibodies for 1 h at room temperature. Images were acquired on the Opera Phenix (PerkinElmer) with the confocal 63× water objective. Immunofluorescence in RPTE cells was performed as described using the above-mentioned antibodies and imaged on the Invitrogen EVOS FL Imaging System (72).

### siRNA and expression construct transfection.

siRNAs targeting RB1 (J-003296-23; Dharmacon), RBL1 (SI02629921; Qiagen), and RBL2 (sc-29425; Santa Cruz) and fluorescein-conjugated nontargeting control siRNA (sc-36869; Santa Cruz) were purchased and diluted to a working concentration of 20 µM. A final concentration of 20 nM was used for all targets in RPTECs, and 40 nM was used in MCF10A cells. siRNAs were delivered to cells using Lipofectamine RNAiMax (Thermo Fisher) as described previously (73).

### Drug treatment.

Cells were treated with 5 µM nutlin (Sigma) for 24 h, and after 18 h of treatment, 25 ng/ml phorbol myristate acetate (PMA; Sigma) was added for the final 6 h prior to RNA extraction. MCF10A cells were treated only with PMA or DMSO, and RNA was isolated 6 h after treatment. For the palbociclib experiments, MCF10A and MCF7 (p53 WT and low A3B expression); HCC1937 and HCC1395 (low A3B expression); T47D, HCC1954, and Hs578T (intermediate A3B expression); and HCC1599, HCC1143, SUM-225-CWN, and HCC202 (high A3B expression) cells were cultured in 6-well plates (Costar 3516; Corning Incorporated) until 70% confluence. Palbociclib (S1116; Selleckchem) was stored as a 5 mM solution in H_2_O and added to cells at concentrations of 0 µM (H_2_O control), 0.5 µM, and 2.5 µM. No palbociclib was added to cells of the 0-h time point, which instead was transferred to ice prior to RNA isolation. RNA was also isolated at 0, 1, and 4 h post-palbociclib administration (total RNA purification kit 37500; Norgen), and cDNA was synthesized with 500 ng RNA (iScript 170-8891; Bio-Rad). RT-qPCR assays for *A3B* and *CCNE2* were performed using the C1000 Thermal Cycler (Bio-Rad). For pretreatment with PMA (tlrl-pma; InvivoGen; 1-mg/ml stock in DMSO), cells were treated with 0 ng/ml (DMSO) or 25 ng/ml PMA for 3 h, followed by treatment with 0 µM, 0.5 µM, and 2.5 µM palbociclib. RNA was isolated 0, 1, and 3 h after addition of palbociclib and processed as described above. For pretreatment with palbociclib, cells were treated with 0 µM, 0.5 µM, and 2.5 µM palbociclib for 30 min, followed by treatment with 0 ng/ml (DMSO) or 25 ng/ml PMA. RNA was isolated 0, 1, and 3 h after addition of PMA and processed as described above.

### Bioinformatics.

TCGA expression data were downloaded from the Broad GDAC Firehose as of January 2016. Expression correlations against A3B by all other genes in the breast cancer cohort were calculated, and significant correlates were used to determine significant activation or inhibition of upstream regulators using Ingenuity Pathway Analysis (Qiagen). All other Spearman correlations and *P* values were calculated, and heat maps were plotted using the R statistical environment. *P* values were adjusted for multiple comparisons using the Bonferroni correction, and resulting *q* values were reported. Cell cycle data were acquired from http://www.targetgenereg.org/ in May 2018.
